# 

**Published:** 1912-05-15

**Authors:** 


### ANNOUNCEMENTS.

The Oral Hygiene Society of the State of Oklahoma Incorporated. The purpose of this organization is to spread the propaganda of Oral Hygiene all over the state by a system of lectures throughout the rural districts as well as in the city schools.

The ultimate result is to have the subject taught in the schools by placing this branch in the common school curriculum.

There is no membership fee charged, every human being can become a member of this society. This movement is endorsed by all educators.

Some men in the profession are fighting this movement but they are only hurting themselves, the fact is that the great public is in favor of it. It is a christian movement, and every man, woman and child who enlists in this great army becomes a philanthropist. The organization was incorporated Thursday, March the 28th, and already has a membership of over five hundred. By June the first we intend to have a membership of more than five thousand. Our place of meeting will be in Oklahoma City, Oklahoma. Literary contributions and donations in cash should be sent to

Miss Libby Goldman, *Secretary and Treas.*,
Lee-Huckins Hotel,
Oklahoma, Okla.

---

The Montana State Board of Dental Examiners will meet in Helena, July 8th, 9th, 10th and 11th, for the regular annual session. All desiring to take the examination will please send in their application to the Secretary thirty days prior to July 8th.

G. A. Chevigny, *Secretary*,
106-8 Clark Block,
Butte, Mont.

---

THE CLINIC OF THE NATIONAL DENTAL ASSOCIATION,

The Clinic Committee desires to extend to all members in good standing of all dental societies a cordial invitation to attend and to clinic at the "all day" clinic of this Association to be held at the New Willard Hotel, Washington, D. C., Friday, September 13th, 1912. The enormous Ball Room, top floor of this hotel has been secured and the manage ment promises us every convenience.

We wish particularly to call your attention to the clas sification of the different clinical material where every effort will be made to arrange the different events according to title and in sequence so that the various phases in any operation may be seen at a glance, without the usual regard to chair or table, this will avoid confusion and save time, allowing the members to select and study favorite subjects without hunting all over the room.

From the material now in hand your committee can promise a large and varied clinic, that we may assemble all clinician's names and titles for the preliminary program, kindly reply at once to

Clarence J. Grieves,
*Chairman Clinic Committee,*
Park Ave. and Madison St.,
Baltimore, Md.

A. O. Ross, *Vice-Chairman,*
807 N. High St., Columbus, O.
S. W. Bowles, *Secretary,*
1616 I St., Washington, D. C.
W. R. Clack, Mason City, Iowa.
A. P. Burkhart, Auburn, N. Y.
J. T. McClenahan, Washington, D. C.
W. D. Tracy, New York City.
George E. Savage, Worcester, Mass.
John H. McClure, Wheeling, W. Va.
J. E. Chace, Ocala, Fla.
E. L. Pettibone, Cleveland, O.
H. J. Allen, Washington, D. C.
W. R. Wright, Jackson, Miss.
C. A. Lundy, Los Angeles, Cal.
S. H. McAfee, New Orleans, La.
C. M. Barnwell, Atlanta, Ga.
Richard L. Simpson, Richmond, Va.
W. H. Scherer, Houston, Tex.

Panama Pacific Dental Congress—Committee on Organization. The first meeting of the Committee on Organization of the Panama-Pacific Dental Congress, which will be held in San Francisco, in 1915, took place in San Francisco on the morning of March 14th, with delegates from practically all the Pacific Coast States present.

The project was entered into with zest and enthusiasm and promises of fullest and widest support were made by all the delegates.

Permanent officers of the committe were elected as follows:

Chairman—Dr. Frank L. Platt.
Secretary—Dr. Arthur M. Flood.
Treasurer—Dr. W. A. L. Knowles.

The Board of Directors will eventually consist of fifteen members. Eight were elected at this meeting, consisting of the Chairman, Secretary and Treasurer of the Committee and five others from the Bay Counties, viz: Drs. H. G. Chappel and J. Loran Pease of Oakland, Dr. F. G. Baird of San Francisco, Dr. R. B. Giffen of Sacramento, and Dr. A. M. Barker of San Jose. The remainder of the fifteen to consist of one from each state participating and one from Southern California, each electing its own Director.

It was voted by the Delegates, that the Board of Directors incorporate as a matter of business sense and expedience. The enthusiasm which prevailed at this meeting promises well for the successful fulfillment of this great project.

As soon as incorporation has been affected, arrangements will be made for enlisting the co-operation and support of every organized dental society in the world.

ARTHUR M. FLOOD, *Secretary.*

---

THE NEW YORK DENTAL MANUFACTURERS EXHIBIT. Dentists to the number of four to six thousand per day visited the great exhibit given by the Dental Manufacturers Club of the United States at 71st Regiment Armory, New York City, April 2 to 5, inclusive.

The exhibit was the largest, most complete and interesting and by far the best arranged and conducted of those given by the Club and much credit is due the various committees having the matter in charge. The Floor Committee is especially to be commended.

Twenty-six concerns were represented on the floor by skillfully arranged exhibits and clinics, all attended by capable demonstrators, each of whom did his best to outshine his competitors.

From the opening hour, 10 A. M. each day until the close, near midnight, the immense hall was filled with interested visitors, each of whom returned to his individual task at the close of the exhibit a better and more enlightened dentist, and a broader and stronger man, we hope.

Among so many fine displays it seem discriminating to select any for special comment, so far as merit is concerned, but by "Following the Crowd" it became evident that the three exhibits given by A. C. Clark & Co., Chicago, two of which were clinical, and the elaborate display of tooth-making processes of the Dentist's Supply Co., New York, were centers of unusual interest, as were also the demonstrations of the Elgin Vacuum Casting Appliance given by the Ransom & Randolph Co., at two conspicuous points on the floor.

The exhibit was a most magnificent success in every way, nothing occuring to mar its harmony or to interfere in the slightest degree with the concentrated interest of the visitors.

---

The next meeting of the State Board of Dental Examiners of Arkansas will be held in Little Rock, Ark., June 17th and 18th. All applicants are required to pass an examination to obtain a certificate. Examination fee $15.—E. H. Johnson, D.D.S., *Secretary and Treasurer*, Citizen's Bank Bldg., Pine Bluff, Ark.

The next meeting of the Arkansas State Dental Association will be held in Little Rock, at Marion Hotel, June 19-21st. Irvin M. Sternberg, D.D.S., *Secretary*.

The forty-fourth annual meeting of the Georgia State Dental Society will be held at Americus, Georgia, June 11th, 12th, 13th, 1912. Instructive papers have been secured and the Clinic Committee will unquestionably present a fine list of clinics. A cordial invitation is extended to all ethical dentists.

M. M. Forbes, D.D.S., *Secretary*,
810-811 Candler Bldg.,
Atlanta, Ga.

---

The Indiana State Dental Association will hold its 54th Annual Meeting at Indianapolis, May 21-23, 1912, at the Claypool Hotel.

Otto U. King, *Secretary*.

---

The meeting of the Kentucky State Dental Association will be held in Louisville, May 27, 28, 29, 1912. A special attraction of talented men from out of the State will be upon the program this year and every indication points to the best meeting that has been held for many years. The dentists of Kentucky are especially invited and a cordial invitation is extended to all ethical members of the profession.

---

The next regular meeting of the Michigan State Board of Dental Examiners will be held at the Dental College, Ann Arbor, commencing Monday, June 17, at 8 A. M., and continuing through the 22d. For application blanks and full particulars, address

F. E. Sharp, *Secretary*,

Port Huron, Mich.

The twenty-ninth annual meeting of the Minnesota State Dental Association which will be held in St. Paul, Minn., June 14, 15, 1912, promises to be the largest in the history of the organization. The large clinic and manufacturers' exhibit will occupy the entire top floor of new Lowry Building, the largest and best equipped dental and medical building in the west. For information, address

BENJAMIN SANDY, *Secretary*,
636 Syndicate Building,
Minneapolis, Minn.

The forty-fifth annual meeting of the Tennessee State Dental Association will be held at Memphis, Tenn., in the Business Men's club rooms, June 6, 7 and 8, 1912. The Business Men's Club extend the courtesies of the Club to all the visiting dentists, and the Association invites all ethical dentists to attend.

J. L. MANIRE, *Secretary*.

The next meeting of the Texas State Board of Dental Examiners, for the purpose of examining applicants for a license to practise dentistry and dental surgery in the State of Texas, will be held in Houston, Tex., beginning June 10th, 1912, at 9 A. M. For application blanks and any further information, address,

J. M. MURPHY, *Secretary*,
Temple, Texas.

The Wisconsin State Board of Dental Examiners will convene in Milwaukee, at Marquette University, on Monday, June 24, 1912, at 9 A. M., for examination of applicants to practise in Milwaukee.

W. T. Hardy, *Secretary*,
422 Jefferson Street,
Milwaukee, Wis.

---

The Second Annual Convention of the Southern, Atlantic County and Mercer Dental Societies. A Tri-Society Dental Exhibition and Convention will be held at Young's Ocean Pier, Atlantic City, New Jersey, the 19th, 20th and 21st of June, 1912. The three societies which have entered into the arrangement are the Southern Dental, Mercer and the Atlantic County, all of New Jersey, and the official name adopted is "The Second Annual Convention under the Auspices of the Southern, Atlantic County and Mercer Dental Societies."

The convention held at Young's Pier in 1911, by the Southern Dental and Atlantic County Societies, proved successful, and with the assistance of the Mercer Society the convention this year should prove to be even a greater success.

Walter W. Crate, D.D.S.,
*Chairman of Press Committee.*

---

Meeting of the National Dental Protective Association. The annual meeting of the National Dental Protective Association will be held at the Fredonia Hotel, Washington, D. C., May 21, 1912, at 7.30 P. M., for the election of Trustees and the transaction of business.

Richard Summa, *President.*
M. F. Finley, *Secretary.*

